# Calciphylaxis Case Series: A Late Presentation of Chronic Kidney Disease From the Eastern Caribbean

**DOI:** 10.7759/cureus.34082

**Published:** 2023-01-23

**Authors:** Amit Ramrattan, Emile P Mohammed, Abigail Cumberbatch, Jeanine Reemaul

**Affiliations:** 1 Internal Medicine, Port-of-Spain General Hospital, Port-of-Spain, TTO; 2 Internal Medicine and Nephrology, Port-of-Spain General Hospital, Port-of-Spain, TTO; 3 Dermatology, Port-of-Spain General Hospital, Port-of-Spain, TTO; 4 Internal Medicine and Dermatology, Port-of-Spain General Hospital, Port-of-Spain, TTO

**Keywords:** treatment of calciphylaxis, calciphylaxis complications, sodium thiosulfate, chronic kidney disease (ckd), calcific uremic arteriopathy

## Abstract

Calcific uremic arteriolopathy (CUA) or calciphylaxis is a rare condition that predominantly affects the dialysis population and is characterized by calcification of cutaneous arterioles accompanied by painful necrotic skin ulcers. At the hemodialysis unit of the Port-of-Spain General Hospital, there have been eight cases between the years 2015-2019 with an incidence of 121 cases per 10,000 patients undergoing renal replacement therapy, quite possibly one of the highest in the world along with an 87.5% mortality when diagnosed with this condition. Risk factors identified in this case series include female gender, obesity, and late presentation of end-stage renal disease. This case series highlights limitations in the diagnosis and management of the disease in a resource-limited setting and intends to raise awareness of this condition in the Caribbean.

## Introduction

Since its first introduction into medical literature by Selye et al in 1961 [1], CUA still carries high morbidity and mortality because of the rapidly progressive nature of the disease process and its therapeutic challenge. It is a disease that largely affects the dialysis population with treatment options that are not evidence-based given its rarity and complexity and reduces the quality of life of the affected patient. Morbidity is related to the fact that ulcerations are non-healing, and painful, and lead to recurrent hospitalizations in an attempt to overcome progressive disease and overwhelming sepsis in most cases. This case series, which is the first for the Eastern Caribbean, highlights eight cases between the years 2015-2019 at the Port-of-Spain General Hospital, Trinidad and Tobago. It raises the possibility of a higher incidence, compared to international statistics, along with underlining the late presentation of CKD as a possible risk factor to the etiology of the disease. The aim of this case series is to assess the prevalence and incidence per 10,000 dialysis patients in Trinidad and Tobago, its mortality at the hemodialysis unit at the Port-of Spain General Hospital of Trinidad and Tobago and to raise awareness of this disease in the Caribbean as it is still under-recognized.

## Materials and methods

Study design

This article is a case series comprising eight patients between the years 2015-2019 with calciphylaxis.

Inclusion and exclusion criteria

Between the years 2015-2019, all patients with renal impairment and skin conditions were reviewed by both Nephrology and Dermatology teams at the Port-of-Spain General Hospital. The highlighted eight cases in this paper were chosen as they met any of the following criteria: history of a rapidly progressive skin ulcer, X-ray findings suggesting the disease, or a skin biopsy suggesting the disease. All patients chosen had renal impairments, such as newly diagnosed chronic kidney disease (CKD), established CKD, or were on renal replacement therapy (RRT). No case presentation with confirmed or suspected calciphylaxis was omitted in this case series.

Data collection

Patients that were hospitalized with calciphylaxis had their case files reviewed. Demographics, case history, investigations, and outcomes were recorded and compiled as case presentations that will be highlighted in the results.

## Results

Case 1

**Figure 1:**
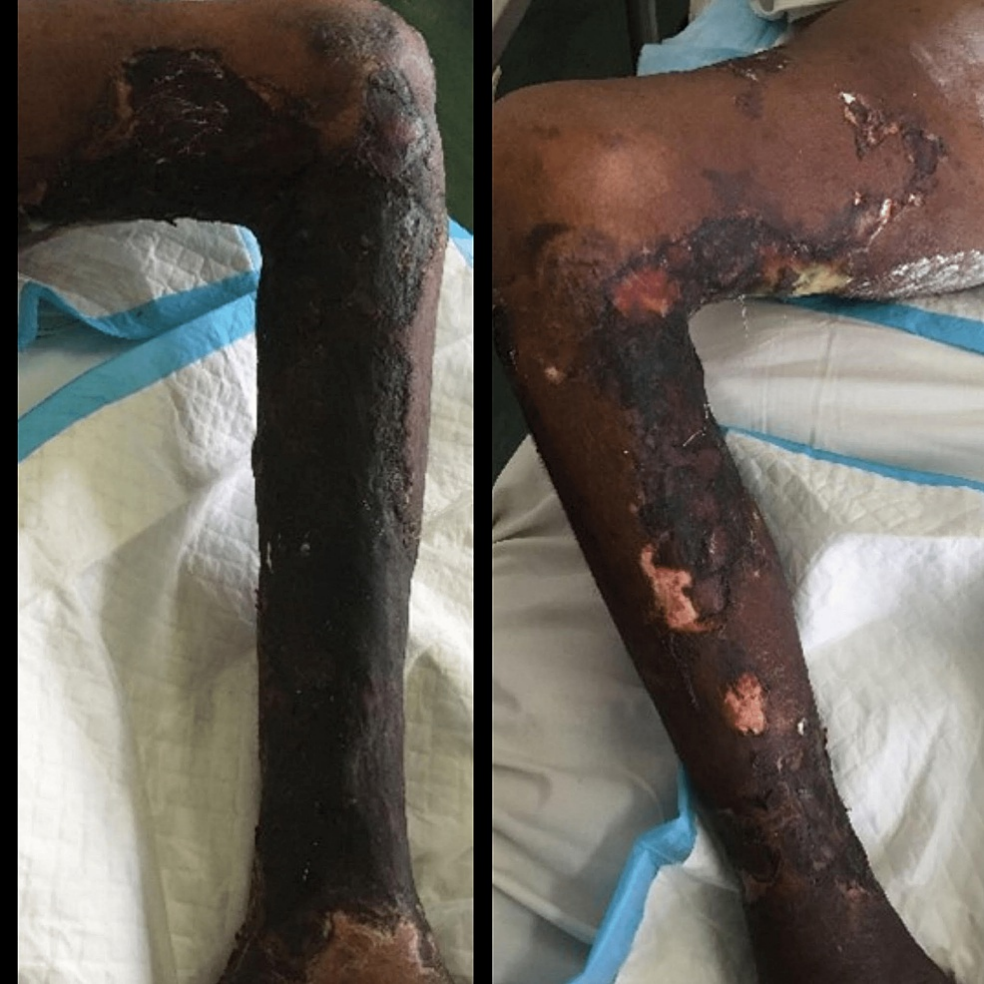
Case 1 - lower limbs The picture depicts both the right and left lower limbs of the patient with widespread necrotic ulcerations.

**Figure 2:**
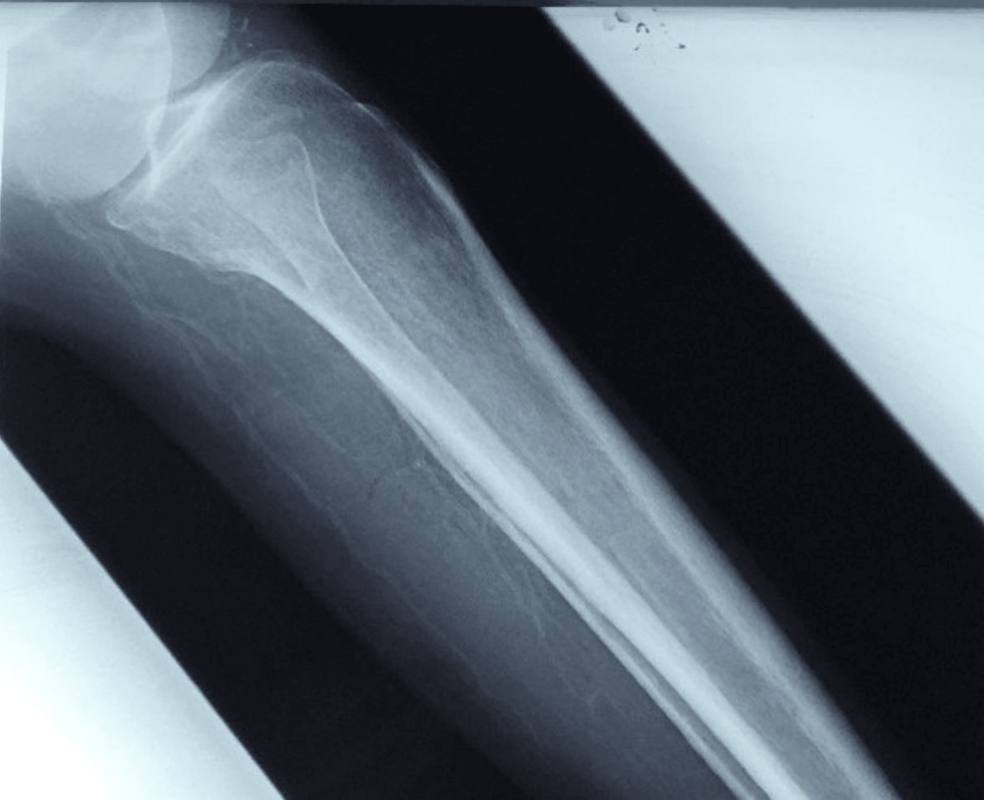
Case 1 - X-ray of right lower limb This X-ray demonstrates calcification of the deep and superficial vessels within the posterior soft tissues.

**Figure 3:**
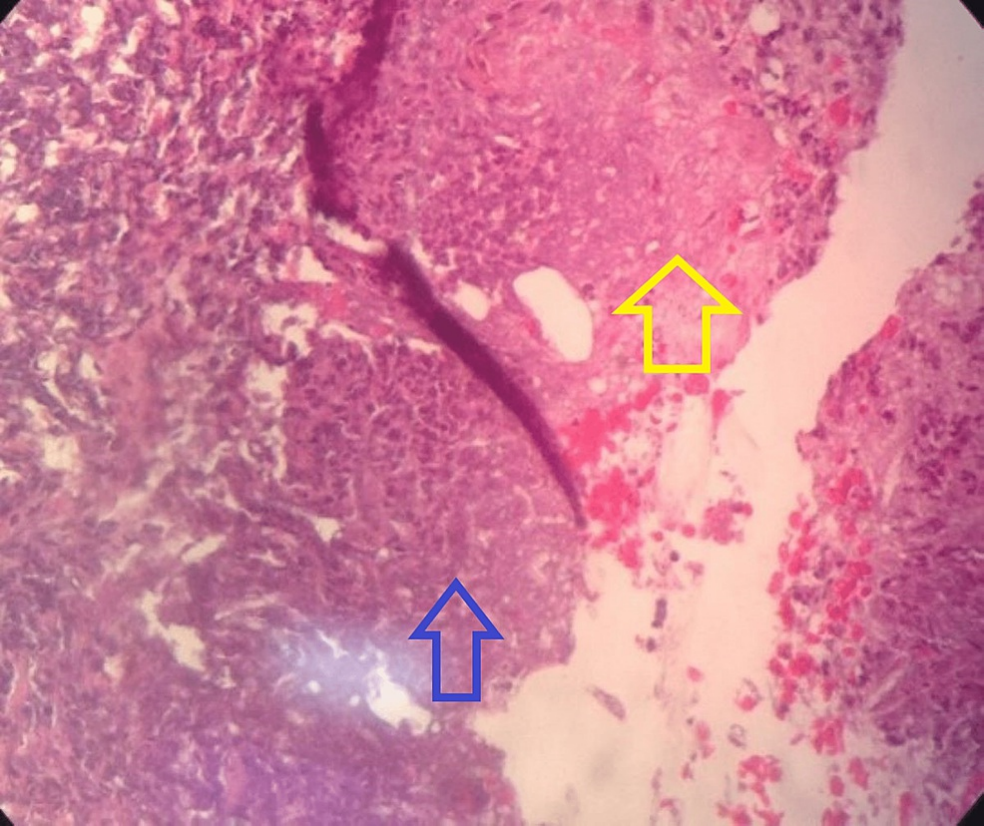
Case 1 - skin biopsy Fibrinoid exudates within the capillaries (yellow arrow) along with numerous neutrophils within the vessels and the adjacent dermis (blue arrow).

A 43-year-old male of East Indian ethnicity, with known diabetes mellitus, hypertension, chronic kidney disease (CKD), and congestive cardiac failure (CCF), presented with a two-week history of lower limb swelling and pain. He had a preceding small blister in his lower limb that rapidly progressed to bilateral extensive hyperpigmented painful and necrotic plaques (Figure 1). An X-ray of the patient's lower limb was arranged as displayed in Figure 2 and his lab investigations are shown in Table 1. This patient was then suspected to have calciphylaxis after which a skin biopsy was performed; the histology slide is shown in Figure 3.

**Table 1:** Lab Investigations This table illustrates the lab results for cases 1 to 8.

Column1	Case 1	Case 2	Case 3	Case 4	Case 5	Case 6	Case 7	Case 8	Normal range
White cell count (x10/L	12.1	14.6	16.4	18.3	14.2	12.2	18.8	13.3	4-10 x10/L
Hemoglobin (g/L)	7.8	9.4	8.4	5.9	3.3	9.7	7.8	9.6	12-15 g/L
Platelet (x10/L)	324	399	453	483	533	317	414	512	150-400 x10/L
Blood Urea Nitrogen (mg/dl)	161		87	210	146		70		6-23 mg/dl
Creatinine (mg/dl)	12.1		16.81	13.06	12.23		7.58		0.5-0.9 mg/dl
ESR (mm/1st hour)	140			140					0-20 mm/1st hour
Calcium (mg/dl)	6.5	8.4	8.2	7.1	8.2	8.9	unavailable at the time	unavailable at the time	8-10 mg/dl
Parathyroid Hormone (pg/ml)	415	unavailable at the time	unavailable at the time	187.4	517	5000	unavailable at the time	1007	10-55 pg/ml
HIV	negative	negative	negative	negative	negative	negative	negative	negative	
Hepatitis B surface antigen	non-reactive	non-reactive	positive	non-reactive	non-reactive	non-reactive	non-reactive	non-reactive	
Hepatitis C Antibody	non-reactive	non-reactive	non-reactive	non-reactive	non-reactive	non-reactive	non-reactive	non-reactive	
Anti-nuclear factor	negative	negative	weakly positive		negative	negative			
Other	p/c ANCA negative	Rheumatoid Factor negative	p/c ANCA negative						0

This patient was commenced on hemodialysis (HD) only thrice weekly at the time due to limitations in the availability of dialysis machines at POSGH. HD continued for approximately five weeks during which he had intermittent temperature spikes with negative line and blood cultures. Wound swabs, however, grew Acinetobacter and Pseudomonas organisms which were treated with intravenous antibiotics. Unfortunately, he succumbed to a massive upper gastrointestinal bleed.

Case 2

**Figure 4:**
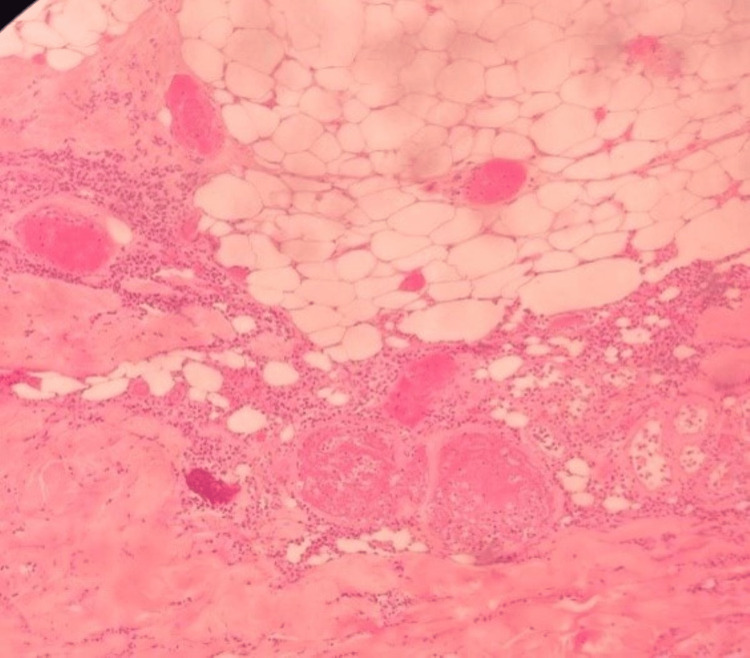
Case 2 - skin biopsy Degenerative and ischemic changes of the skin ulcer with a florid acute vasculitis of the medium and small vessels, as well as intraluminal calcification, suggestive of Calciphylaxis

A 48-year-old female Afro-Caribbean patient with known CKD on HD three times weekly for over one year presented with a rapidly worsening and painful rash on her lower limbs. On assessment, she had eschars on her thighs in addition to hyperpigmented ulcerated lesions on both lower limbs with extension to the toes. The patient was suspected to have calciphylaxis. X-rays of her lower limbs done at the time showed numerous vascular calcifications within the soft tissues and the popliteal and posterior tibial arteries. Blood investigations are highlighted in Table 1. A skin biopsy was then arranged as shown in Figure 4.

The patient was transitioned to daily HD treatment. She underwent daily changes of dressings and was treated for possible Pseudomonas aeruginosa infection with piperacillin/tazobactam. Sodium thiosulphate was unavailable at this time to possibly treat the disease and the patient regrettably deteriorated and died from sepsis within one month after her diagnosis.

Case 3

**Figure 5:**
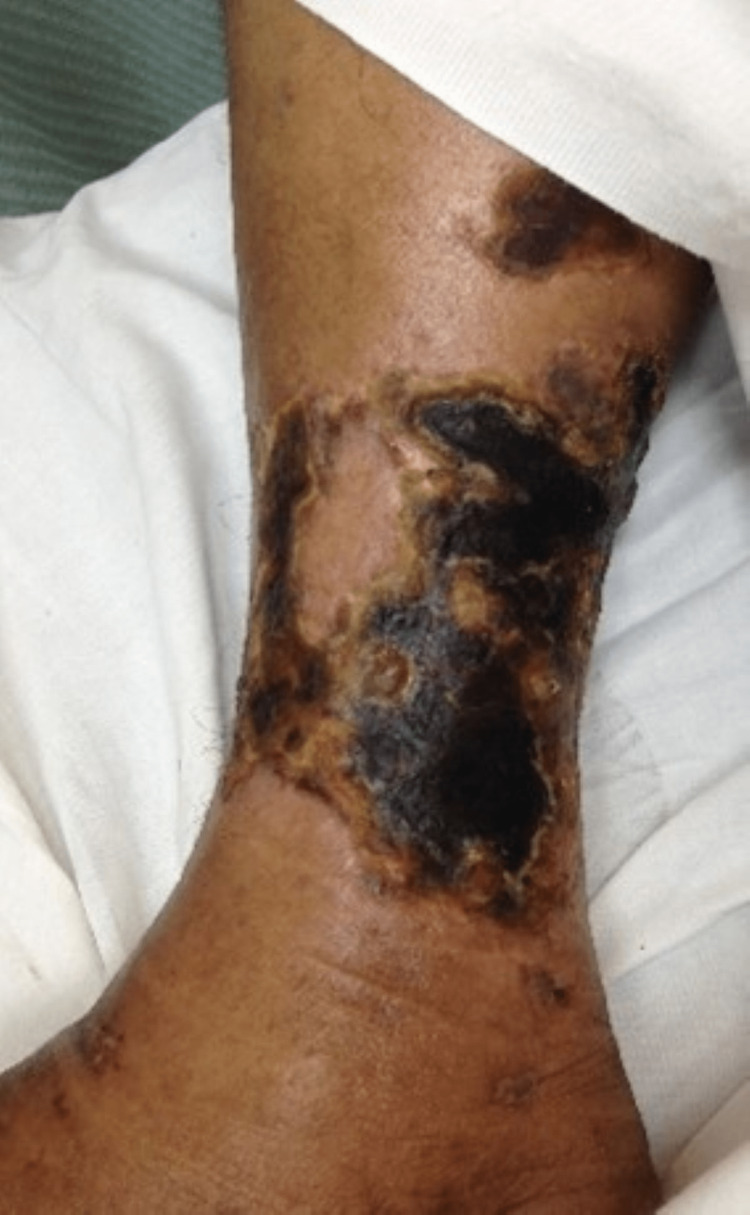
Case 3 - lower limb lesion This picture depicts the patient's necrotic skin ulceration.

A 46-year-old obese male of mixed ethnicity, who had a known history of CKD secondary to hypertension with follow-up at the Nephrology Outpatient Clinic (NOPC) POSGH, was admitted under the general surgery team for lower limb cellulitis. Three weeks prior, he gave a history of the appearance of a hyperpigmented spot which subsequently developed into a blister and then ulceration. This rapidly progressed into hyperpigmented necrotic painful plaques illustrated in Figure 5.

**Figure 6:**
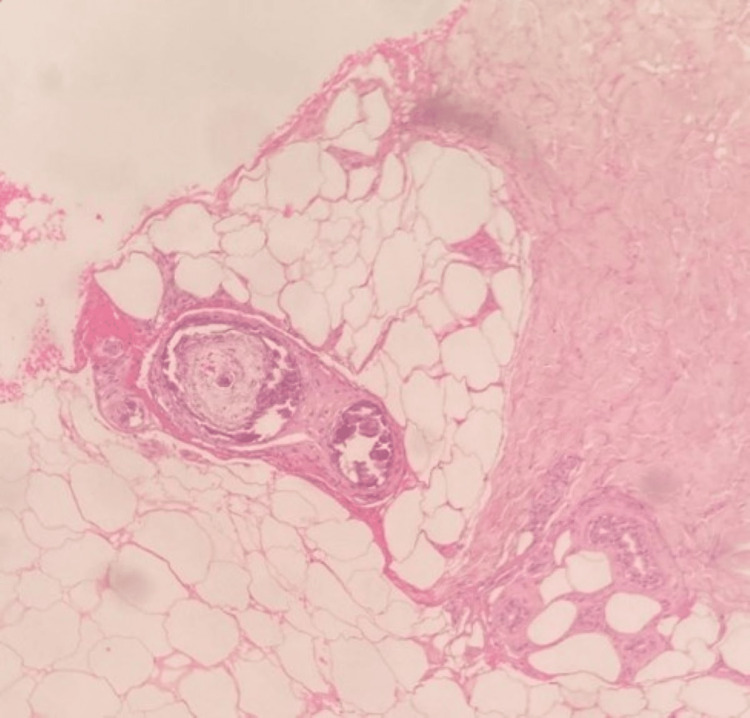
Case 3 - skin biopsy Epidermonecrosis with intraepithelial bullous formation, focal fibrinoid exudates in the superficial vessels and focal calcification of the vessels deep in the subcutis suggestive of calciphylaxis.

Lab investigations are highlighted in Table 1 but most notably, his Hepatitis B surface antigen test was positive. With a clinical suspicion of calciphylaxis, X-rays performed on his lower limb showed vascular calcification deep to the Achilles tendon and a skin biopsy was arranged as shown in Figure 6. 

During this time, he was placed on ceftazidime for possible Pseudomonas infection of this ulcer and had daily changes of dressings. POSGH, at that time, had no facilities to offer HD for hepatitis-positive patients, thus, a peritoneal dialysis (PD) catheter was inserted for RRT. Peritoneal dialysis, however, could not have been initiated in a timely manner and there was no availability of sodium thiosulphate. With the progression of his calciphylaxis in addition to worsening uremia, the patient succumbed to his complications within one month of the diagnosis.

Case 4

A 74-year-old obese female of East Indian ethnicity, hypertensive and with known diabetes mellitus, presented to POSGH with shortness of breath, lower limb swelling, and melena stools with lab investigations as per Table 1. On examination, the patient was confused, possibly due to uremic encephalopathy versus delirium from sepsis, but most notably, she had extensive necrotic plaques in her lower limbs. Discussion with her family members elicited a history whereby the patient was bitten by mosquitoes two weeks prior where the initial hyperpigmented macules on her lower limbs had rapidly progressed to necrotic ulcerations on this admission. The priority at this time was to commence HD on the patient and investigate for a possible upper gastrointestinal bleed. An upper endoscopy performed had mild gastritis and duodenitis, likely due to uremia, but no ulcerations or bleeding to account for her melena stool. The patient, after a week of initiating daily HD, succumbed to severe sepsis before investigations could have been arranged to confirm the clinical suspicion of Calciphylaxis, which was the likely diagnosis in this case given the presentation.

Case 5

**Figure 7:**
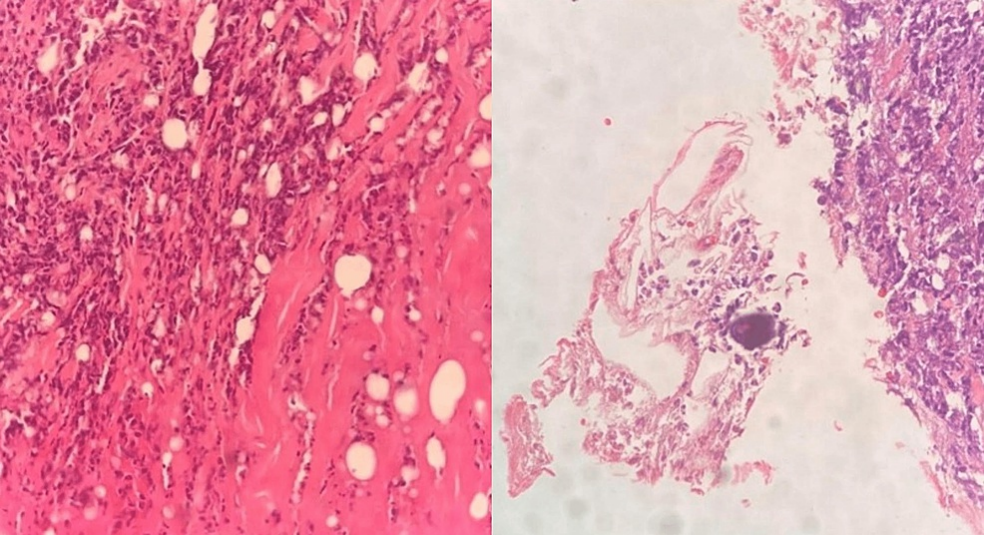
Case 5 - skin biopsy Ulcerative necrosis of the epidermis and upper dermis with fibrinoid exudates within the vessels and a heavy inflammatory infiltrate in the mid to deep dermis with calcified material within the moderate size vessel consistent with calciphylaxis.

A 58-year-old female of Afro-Caribbean ethnicity, who had known CKD secondary to hypertension with no follow-up at the NOPC POSGH for two years, presented to the hospital with symptoms of anemia. Her lab investigations can be seen in Table 1. The patient was, however, found to have painful necrotic ulcerations in her lower limbs that had rapidly progressed from simple mosquito bites and blisters three weeks prior. The patient was transfused and commenced on HD via right internal jugular vein permanent catheter whilst investigations were done. X-rays of her lower limbs showed widespread calcification of the superficial femoral, popliteal, posterior and anterior tibial arteries. A skin biopsy was done as shown in Figure 7.

The patient was commenced on daily HD in an outpatient setting but there was no sodium thiosulphate available to treat the condition. Hypoglycemia, sepsis, and hypotensive episodes in dialysis then ensued which penultimately led to recurrent hospital admissions. The patient eventually succumbed to her complications, no more than three months after her diagnosis.

Case 6

**Figure 8:**
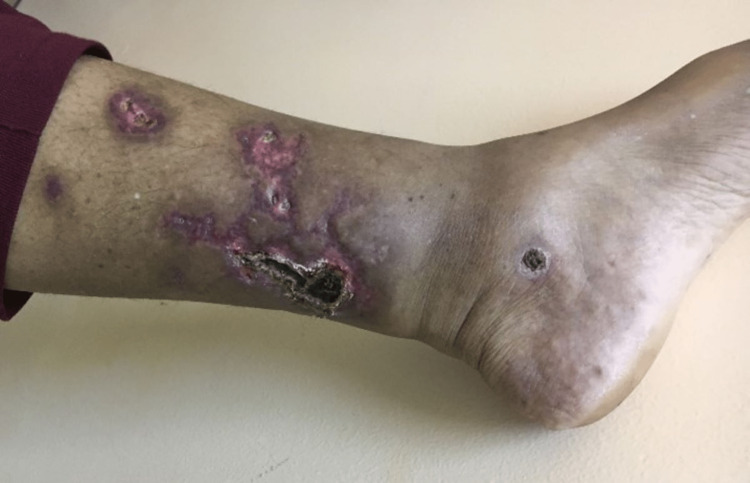
Case 6 - skin lesion This picture demonstrates a necrotic skin ulceration with erythema and surrounding hyperpigmentation.

**Figure 9:**
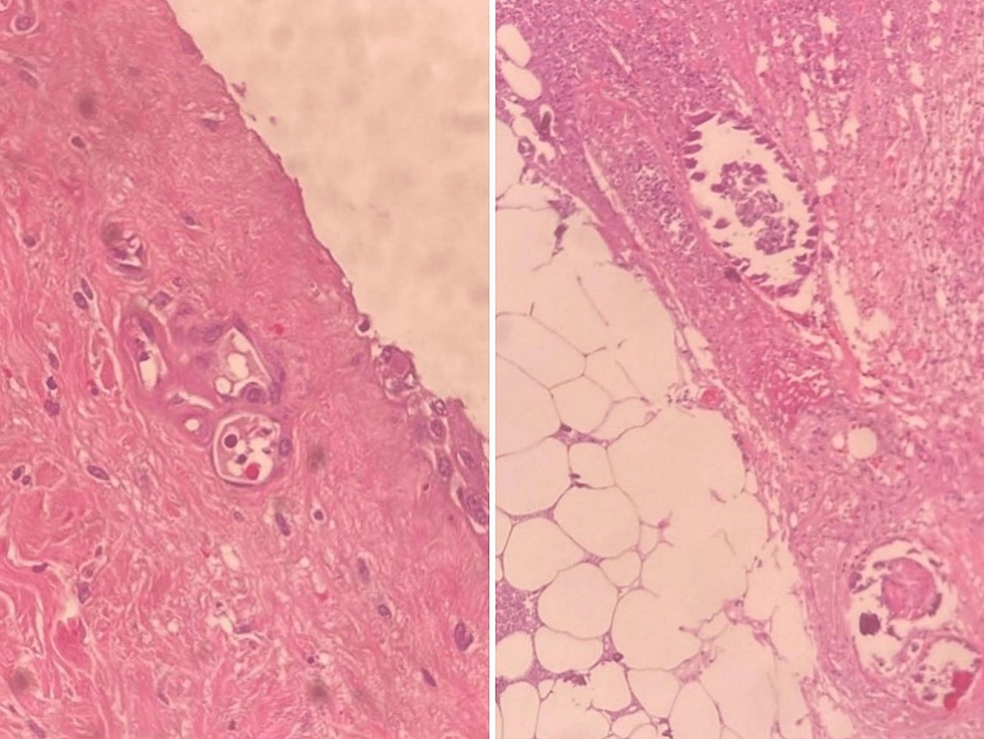
Case 6 - skin biopsy Extensive fat necrosis with histocytes and scattered neutrophils within the dermis and small and medium sized vessels containing calcium deposits, but fibrin thrombi were not easily identified

A 49-year-old obese female of East Indian ethnicity, who was known CKD secondary to hypertension, presented to a private institution where she was suspected to have calciphylaxis. She was commenced on HD at that time and was referred to the POSGH NOPC for follow-up as well as the Dermatology Outpatient clinic for further investigations and management. When reviewed in the clinics, a history elicited that one month prior to her commencement of HD, she had intensely painful necrotic ulcers which were rapidly progressing (Figure 8). Skin biopsy was done as shown in Figure 9.

**Figure 10:**
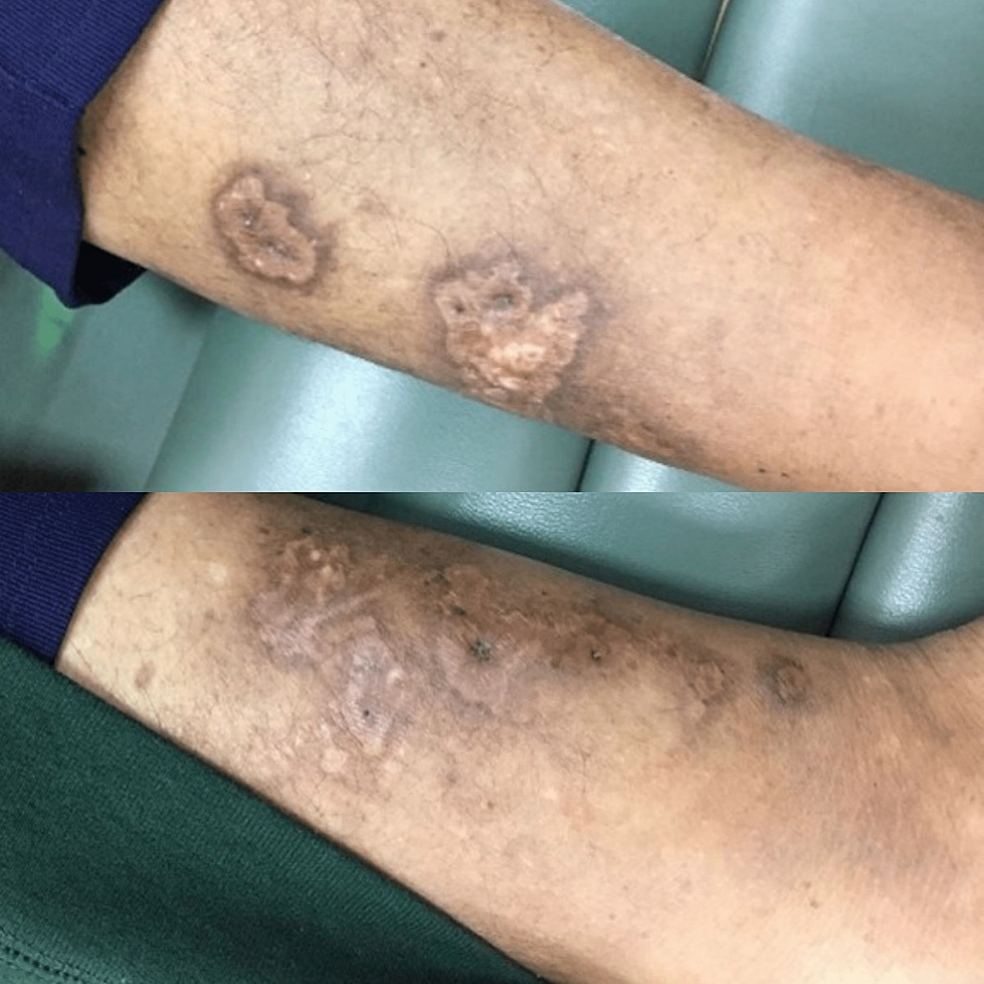
Case 6 Healing Skin lesions When compared to figure 8, the patient's skin ulceration has healing with likely post-inflammatory hyperpigmentation.

The skin biopsy raised the possibility of calciphylaxis, and the patient’s HD was increased to daily at POSGH. Sodium thiosulphate was available at this time and the patient was started at 50g at HD three times for the first week and continued at 25g thrice weekly at HD for the next eight months. Lab investigations can be seen in Table 1 but notably, the patient had a parathyroid hormone (PTH) level over 5000pg/ml after which Rocaltrol was commenced. She was referred to the Ears Nose and Throat Clinic for possible parathyroidectomy. The patient subsequently underwent a right hemithyroidectomy given difficulty intraoperatively to locate the parathyroid glands. Her PTH post-op had dropped to 2261pg/ml. During this time the patient was still receiving sodium thiosulphate and her skin lesions had healed significantly with resolution to her intense pain (Figure 10).

After eight months of receiving sodium thiosulphate, it was discontinued due to an absence of supply at hospital. However, to date there has been no recurrence of the lesions. The patient is currently on paracalcitriol for her gyperparathyroidism as she had opted out of further surgical intervention and to date is well.

Case 7

**Figure 11:**
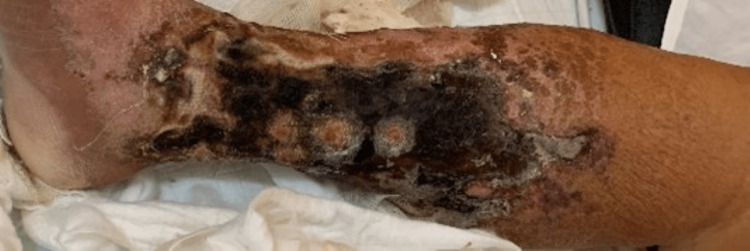
Case 7 skin lesion This picture demonstrates the patient with lower limb necrotic ulcerations and plaques from mid to lower calf and distal desquamation of the skin.

A 48-year-old female of mixed ethnicity with known CKD secondary to diabetes mellitus and hypertension stage 5 in NOPC POSGH presented to the hospital for management of venous ulcers. Her past history revealed a blister three months prior to presentation in her right lower limb which then progressed and extended proximally and to the left lower limb to necrotic ulcers (Figure 11) that were painful and had a foul odor and discharge.

**Figure 12:**
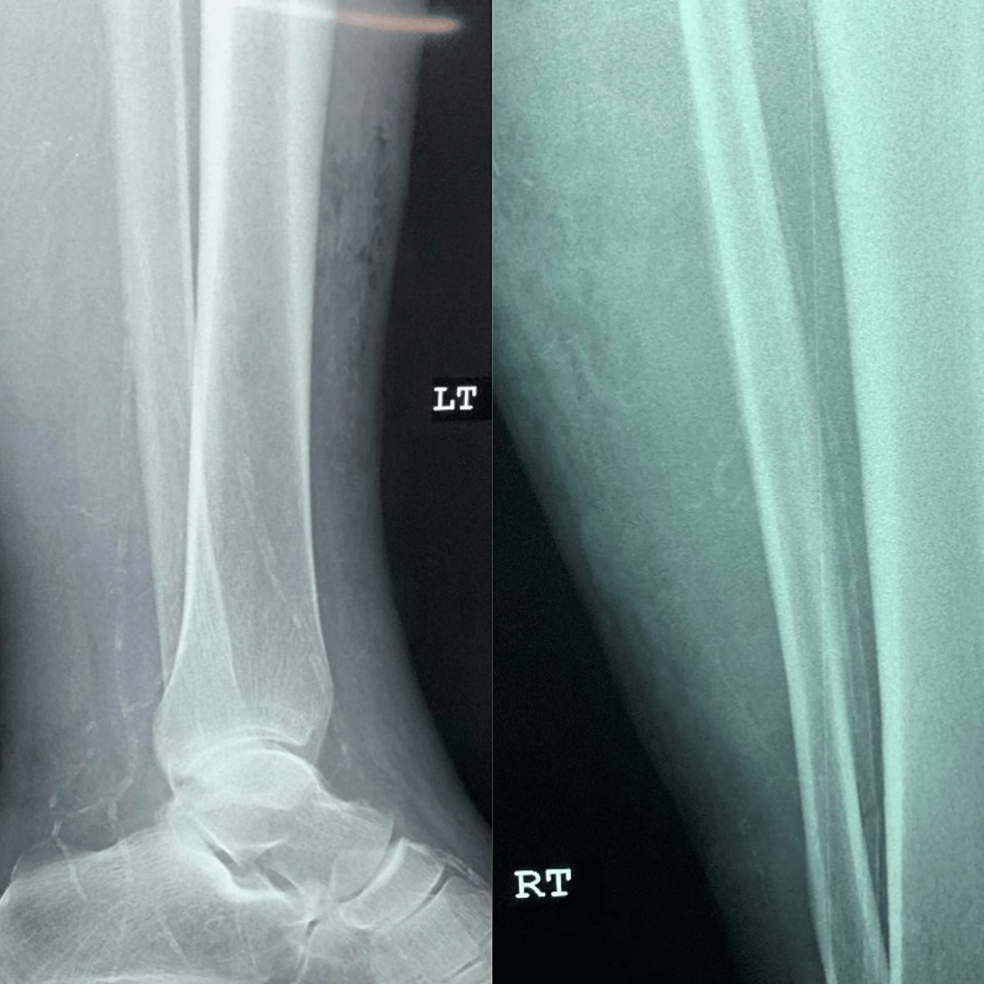
Case 7 - X-ray of lower limbs Both lower limb X-rays demonstrate calcification of the medium and small vessels as well as the subcutaneous tissue, especially in the right lower limb

**Figure 13:**
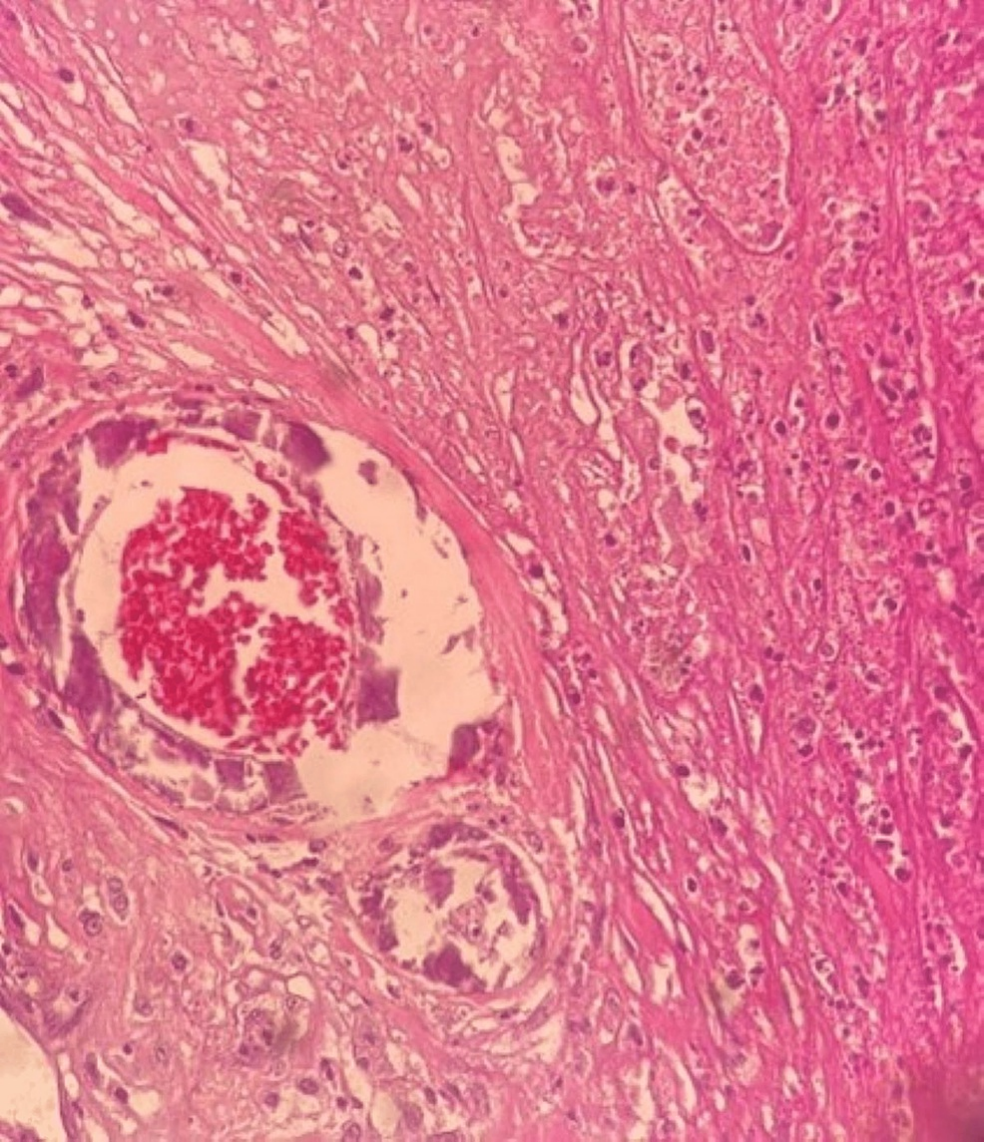
Case 7 - skin biopsy Ulceration of the epidermis with severe acute inflammation and necrosis with marked calcification and thrombi within the small and intermediate vessels of the subcutaneous tissue in keeping with Calciphylaxis

The patient's lab investigations are illustrated in Table 1. A wound swab of the ulcer grew Pseudomonas and E. Coli. The patient was subsequently treated with piperacillin/tazobactam. X-rays were arranged for suspected calciphylaxis (Figure 12) and along with a skin biopsy (Figure 13).

The patient had a right internal jugular vein permanent catheter placed and was commenced on daily HD. There was no sodium thiosulphate available for this patient and after some time, the patient succumbed to her condition and severe sepsis no more than three months after her diagnosis.

Case 8

**Figure 14:**
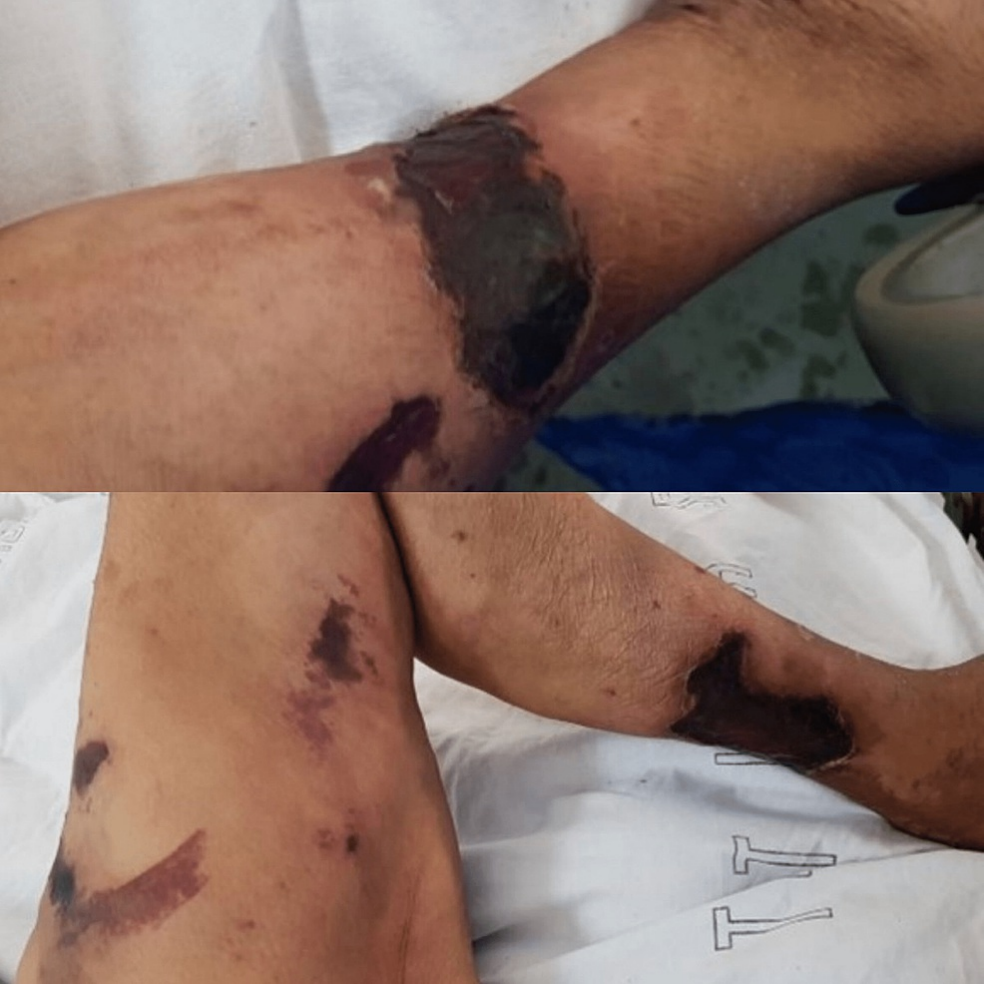
Case 8 - lower limb skin lesions This picture depicts necrotic ulcerations in both lower limbs with the left lower limb having similar ulcerations in the proximal leg.

The patient was a 42-year-old female, obese and of mixed ethnicity, had known CKD secondary to hypertension vs. primary glomerulonephritis, and was on HD for approximately three years. She presented to the nephrology team for rapidly worsening and painful leg ulcerations (Figure 14).

**Figure 15:**
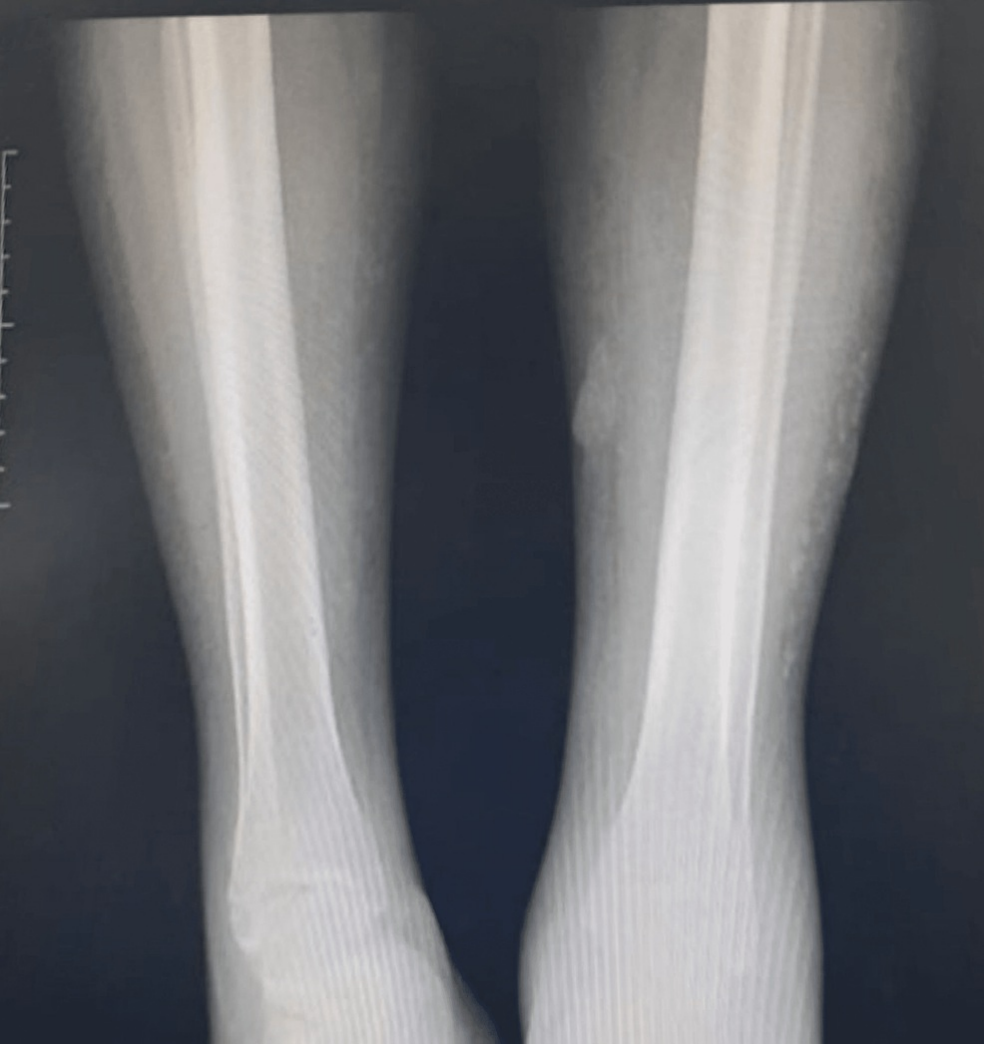
Case 8 - X-ray of lower limbs Evidence of bilateral subcutaneous ankle edema with lower limb vessel calcification and soft tissue calcification suggesting calciphylaxis.

**Figure 16:**
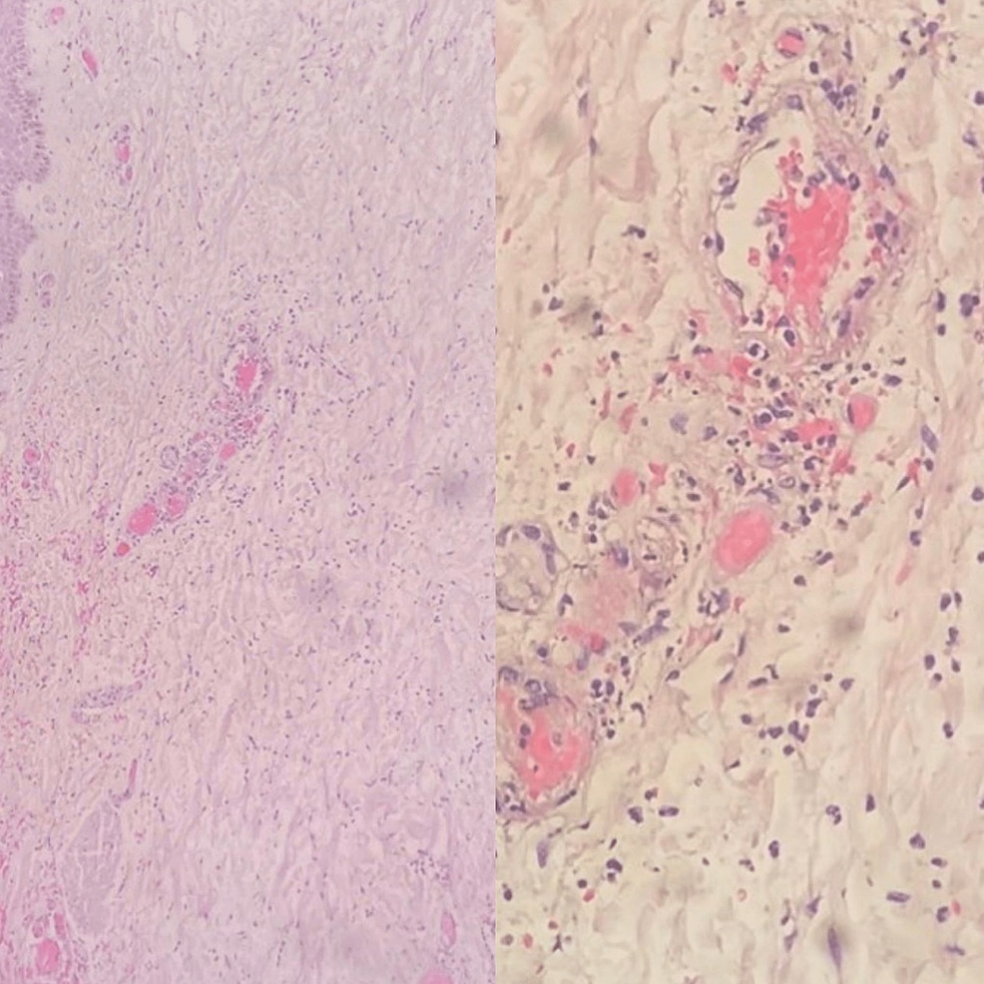
Case 8 - skin biopsy No findings of fat necrosis or calcifications, but there was evidence of marked neutrophilic inflammation suggesting a leukocytoclastic vasculitis. Clinical correlation advised.

The patient was suspected to have Calciphylaxis at this time and her dialysis was increased to daily whilst investigations were done. X-rays of her lower limbs were done (Figure 15), and a skin biopsy was subsequently performed (Figure 16). 

At the time, there was no sodium thiosulphate available for possibly treating the condition and the patient deteriorated and succumbed to severe sepsis before further work-up could have been done.

## Discussion

This rare cutaneous-systemic small vasculopathy is characterized by mural calcification, intimal proliferation, fibrosis, and thrombosis. This condition occurs largely in end-stage renal disease (ESRD) with an incidence of 35 cases per 10,000 patients undergoing HD in the United States, 4 per 10,000 in Germany, and less than 1 per 10,000 in Japan. The manifestation of calciphylaxis occurred approximately 30 months after the initiation of dialysis in the US and Germany [2].

This, however, is not demonstrated in this case series as six out of our eight presented cases had dialysis initiated only after the presentation of calciphylaxis. In an oral presentation presented at the Northwest Regional Health Authority Research Day 2018 [3], 265 patients were commenced on RRT between 2016-2017. This suggests an average annual incidence of 132 new patients. Given there were eight CUA cases over a five-year period at the POSGH, this suggests an incidence of 121 cases per 10,000 patients undergoing RRT, which could be one of the highest in the world. CUA carries a one-year mortality of 45-80% with ulcerated lesions compared to nonulcerated lesions [4] with sepsis as the leading cause of death, but this case series, however, showed a higher mortality of 87.5% within three months of diagnosis. The lesions of CUA mimic other ischemic dermopathies such as warfarin-induced skin necrosis and necrotizing fasciitis [5]. These lesions are painful at presentation with rapid progression to malodorous necrotizing ulcers with overlying black eschars. The pain was notably a significant cause of morbidity throughout the disease course.

The pathogenesis of this condition remains largely uncertain and is beyond the scope of this case series. What is known, however, is that this condition in the ESRD population is associated with hypercalcemia, hyperphosphatemia, elevated PTH, and hypoalbuminemia [6]. Additional identified risk factors include obesity, female sex, and diabetes. In this case series, 75% of the cases were female patients receiving hemodialysis and four of the eight cases were obese, thus mirroring these associated risk factors that have been identified in other studies. Since six out of the eight cases had calciphylaxis before the commencement of dialysis, one can now question whether a late presentation of ESRD may be a risk factor for an earlier onset of calciphylaxis in addition to being a poor prognostic indicator associated with higher mortality.

Management of this condition, with an overall poor prognosis, is multifactorial and is aimed at reducing modifiable risk factors and promoting cutaneous wound healing. Increasing the frequency of dialysis was the first management option implemented in these cases as it has been shown to promote wound healing [7]. Avoiding hypercalcemia and hyperphosphatemia is the next goal that can be achieved with intense dialysis regime and low calcium in the dialysate. The novel drug, sodium thiosulphate, is now creating favorable outcomes that have been reported including rapid resolution of pain, improved wound healing, and improvement in one-year mortality [8]. This was highlighted in Case 6 of this case series. Sodium thiosulfate is thought to be effective in treating calciphylaxis by several mechanisms, including antioxidation, vasodilation, and chelation [9,10]. Due to the unavailability of medications to effectively manage secondary hyperparathyroidism, this patient also underwent a parathyroidectomy, which has suggestive benefits with long-term survival and the promotion of wound healing [11].

Wound management plays a pivotal role in the management of calciphylaxis with the aim of preventing infection, promoting wound healing, and keeping wounds free of necrotic tissue. Though surgical debridement is controversial, a retrospective analysis from Mayo Clinic [12] showed a one-year survival rate of 61.6% for patients who underwent surgical debridement compared to 27.4% for those who did not. With sepsis being the leading cause of death, infected calciphylaxis should prompt surgical consultation whereas non-infected, dry, and stable calciphylaxis should be managed with chemical debridement [13].

There were undoubtedly limitations in this case series. Due to the debilitated status of the patients, it was not possible to perform X-rays and/or skin biopsies which would have solidified their diagnoses. In a resource-limited setting, PTH, calcium, and phosphorus tests were not available for all cases. Thus, if hyperparathyroidism was a contributing factor, this could not have been detected or treated appropriately. Furthermore, the lack of adequate wound care led to worsening sepsis and death in patients. The input of specialized burns or plastic teams may have delayed or even altered the outcome of these patients.

## Conclusions

This case series highlights eight cases of calciphylaxis encountered during the period 2015-2019 at the Port-of-Spain General Hospital. In a resource-limited setting, management of this condition is undoubtedly challenging as highlighted by the high mortality rate. Due to a lack of a National Registry for both dialysis and calciphylaxis patients, the true incidence cannot be determined but one can objectively conclude that given our population size, the incidence is much higher compared to international rates despite the rarity of this disease. There should therefore be a concerted effort towards greater awareness of this condition, not only in Trinidad and Tobago but also throughout the Caribbean, as prompt recognition of this condition with early, vigorous treatment could largely improve mortality. A multidisciplinary and multi-interventional approach is advised in managing these patients with input from nephrology, dermatology, nutrition, general surgery, and otolaryngology teams.
